# RETRACTION: Modern Techniques in Addressing Facial Acne Scars: A Thorough Analysis

**DOI:** 10.1111/srt.70218

**Published:** 2025-08-25

Miao L, Ma Y, Liu Z, Ruan H, Yuan B. Modern techniques in addressing facial acne scars: A thorough analysis. *Skin Res Technol*. 2024;30(2):e13573. doi:10.1111/srt.13573

The above article, published online on 1 February 2024 in Wiley Online Library (wileyonlinelibrary.com), has been retracted by agreement between *Skin Research and Technology* and John Wiley & Sons Ltd. The article was accepted for publication following an insufficient peer review process that does not meet the standards of the journal's peer review policy. Furthermore, the article includes images obtained from online sources without proper consent and authorization for use. As a result, the article has been retracted. The authors have been informed of this decision but unavailable for a final confirmation.

